# High surface area micro-mesoporous graphene for electrochemical applications

**DOI:** 10.1038/s41598-021-01154-0

**Published:** 2021-11-11

**Authors:** Piotr Kamedulski, Malgorzata Skorupska, Pawel Binkowski, Weronika Arendarska, Anna Ilnicka, Jerzy P. Lukaszewicz

**Affiliations:** 1 Faculty of Chemistry, Nicolaus Copernicus University, Gagarina 7, 87-100 Torun, Poland; 2 Centre for Modern Interdisciplinary Technologies, Nicolaus Copernicus University, Wilenska 4, 87-100 Torun, Poland

**Keywords:** Chemistry, Energy science and technology, Materials science

## Abstract

The manuscript presents results on the influence of external pressure on graphene exfoliation and subsequent 3D structuring by means of liquid-phase exfoliation. In contrast to known and applied exfoliation methods, the current study exploits the enhancement of splitting forces caused by the application of high pressure. The manufacturing pathway allowed to increase the surface area from 750 m^2^/g (nanoplatelets) to ca. 1100 m^2^/g (after 3D structuring). Electrochemical studies revealed that the 3D graphene materials were active in the oxygen reduction reaction (ORR). The outstanding ORR activity of 3D structured graphene materials should not be ascribed to heteroatom catalytic centers since such heteroatoms were successively removed upon increasing the carbonization temperature. XPS data showed that the presence of transition metals and nitrogen (usually regarded as catalytic centers) in G-materials was marginal. The results highlight the importance of structural factors of electrodes in the case of graphene-based materials for Zn–air batteries and ORR.

## Introduction

Graphene still attracts researchers’ attention due to some outstanding features, such as high (almost “metallic”) electric conductivity, heteroatom insertion ability, and high chemical stability. The feature might predestine graphene for an ideal electrode material in electrochemical devices like batteries and supercapacitors. However, two important features of common electrode materials cannot be ascribed to pristine, i.e. strictly monolayer graphene: low manufacturing cost as well as a well-developed surface area and pore structure. Although a surface area of 2640 m^2^/g should be theoretically expected from graphene, real graphene samples, mainly nanoplatelets, exhibit a much less developed surface area of 10–750 m^2^/g^1,2^. This fact, along with a relatively high price of typical 2D graphene^3^, turned researchers’ attention to other sources of graphene, i.e. graphite and its exfoliation to less agglomerated structures. Several exfoliation methods were reported, such as liquid-phase, electrochemical, mechanical and microwave^4–12^. Liquid-phase exfoliation is regarded as a scalable method yielding graphene flakes of high quality and purity and low agglomeration^13^. The quality of such exfoliated graphene sheets may be as high as for graphene sheets obtained by chemical vapour deposition or SiC-based synthesis. However, liquid-phase exfoliation does not solve the problem of the lack of a durable pore structure resulting from the exfoliation process. The interflake spaces, even if created, collapse after the removal of the liquid phase. To avoid this effect, which practically excludes exfoliated graphene from electrode manufacturing, some additional measures must be undertaken. Such procedures should fix the split flakes at a certain distance from each other. Thus, in our previous studies, the liquid phase splitting was supplemented by the 3D structuring of exfoliated graphene flakes^14–16^. 3D structuring employs a nanopowder, of which particles (so-called hard template) penetrate the newly formed spaces between the exfoliated graphene flakes, providing a permanent separation of them and avoiding a secondary stacking due to π–π attracting forces. Polyfurfuryl alcohol was used as “carbon glue” for a durable fixation of the obtained 3D-structured graphene flakes. Although the manufacturing scenario was practised recently, the answer to an important question is still missing: which factors may support the process of spontaneous graphene flake splitting in the liquid phase. Originally, the process relies on the appropriate surface tension of the liquid and the wettability of the graphene surface. Too high surface tension and too low wettability prevent the penetration of the dispersing liquid in-between the stack graphene flakes and hinder their subsequent separation. Thus, a hypothesis was raised that external high pressure set on the dispersing liquid should increase the effectiveness of the whole exfoliation process. The current paper addresses this pressure-exfoliation issue and is intending, as a pioneering study, to prove the positive influence of forced liquid exfoliation of graphene nanoplates to less agglomerated graphene structures, which can be structured to 3D electrode materials.

## Materials and methods

### Sample preparation

Mesoporous graphene was formulated similar to our previously published protocols^17^. In summary, 2 g of pristine graphene nanoplatelets (Sigma-Aldrich 900407, surface area 750 m^2^/g) was added to 4 g of CaCO_3_ (SkySpring Nanomaterials, INC., average particle size of 15–40 nm) and mixed mechanically. Then, 20 ml of 1-methyl-2-pyrrolidinone, NMP (Sigma-Aldrich, 443778) with 0.1 ml of cationic surfactant CTAC was added and mixed well. Next, the flask was placed for 1 h in an ultrasonic bath (room temperature) and then, 15 ml of furfuryl alcohol (POCh) was added, mixed with 3 drops of concentrated phosphoric acid aqueous solutions (75%) in an ARE-250 planetary mixer. Next, everything was poured into a Teflon container and finally placed in a miniclave drive pressure reactor (Büchiglasuster, Switzerland). The temperature (− 0.55 °C) in the reactor was kept constant for 2 h at a pressure of 12 bar. After 2 h, the temperature was increased sharply to 25 °C. Then, it was dried in an electric furnace at 50 °C for 2 h. Next, the material was divided into four samples for carbonization at temperatures in the range of 700–900 °C. The mass was heated under the flow of nitrogen at a rate of 10 °C/min in a tube furnace (Thermolyne F21100). After this process, the prepared samples remained in a tube furnace under the flow of nitrogen in order to reach room temperature. After carbonization, the samples were treated as in our previous works^17,18^ with concentrated (34–37%) HCl for 20 min (1 g of carbon was used per 12 ml of HCl) and then washed with distilled water using a Büchner funnel, until the pH of the solution reached 6–7. The action of HCl opened pores in the carbon matrix by etching the template. Then, it was dried in an electric furnace at 100 °C for 24 h^17,18^. The term 3D graphene is when graphene flakes get stack randomly in a 3D volume^19–24^. Thus, we receive a 3D bulky structure (graphene flakes are building stones) resembling a sponge. “3D Graphene” is only a mental shortcut. The “3D structured graphene flakes” is a better description, however too long to be commonly permanently, and therefore we consequently use shorter forms in the text. The three-dimensional graphene samples obtained in the proposed method were denoted as G-T, where T means the carbonization temperature of 700 °C (G-700), 800 °C (G-800) or 900 °C (G-900). The precursor of 3D materials is called GF-750.

### Methods of analysis

The morphology of the obtained high surface mesoporous graphene was analyzed by scanning electron microscopy with STEM mode (SEM, 1430 VP, LEO Electron Microscopy Ltd., Oberkochen, Germany) and high-resolution transmission electron microscopy (HRTEM, FEI Europeproduction, model Tecnai F20 X-Twin, Brno, Czech Republic). The graphene samples obtained prior to the HRTEM microscopic analysis were dispersed in ethanol and treated with an Inter Sonic IS-1K bath for 15 min and deposited on holey carbon-coated copper grids. The volumetric elemental composition (C, N, H) of the materials was analyzed by means of a combustion elementalanalyzer (Vario MACRO CHN, Elementar Analysensysteme GmbH, Langenselbold, Germany). The structural properties and nitrogen sorption isotherms of obtained graphene materials were determined by nitrogen physisorption experiments at − 196 °C using an ASAP 2020 Plus instrument (Micromeritics, USA). The surface area was calculated by the Brunauer–Emmett–Teller (BET) model. The pore size distributions were determined from the nonlocalized density functional theory (NLDFT) method in the SAIEUS program. The hysteresis loop area in adsorption units (cm^3^ g^−1^) was obtained as a result of numerical integration as the difference of the surface area under the desorption and adsorption branches. Raman spectra were obtained by a micro-Raman spectrometer (laser wavelength 532 nm, Senterra, Bruker Optik, Billerica, MA, USA). The laser was tightly focused on the sample surface through a 50× microscope objective. To prevent any damage to the sample, excitation power was fixed at 2 mW. The resolution was 4 cm^−1^, CCD temperature 223 K, laser spot diameter 2.0 μm, and total integration time 100 s (50 × 2 s) were used. X-ray photoelectron spectroscopy (XPS, PHI5000 VersaProbe II Scanning XPS Microprobe, Chigasaki, Japan) measurements were performed using a monochromatic Al Kα X-ray source. Survey spectra were recorded for all samples in the energy range of 0–1300 eV with a 0.5 eV step, high-resolution spectra were recorded with a 0.1 eV step. The electrochemical studies of the oxygen reduction reaction (ORR) and Zn–air batteries measurements were carried out with a potentiostat (Autolab, PGSTAT128N, Netherland) and were performed according to methods published in our other article^14,17^.

### Electrochemical measurements ORR

The electrochemical studies of the oxygen reduction reaction (ORR) were carried out with a potentiostat (Autolab, PGSTAT128N, Netherland). The tests were performed in a three-electrode system, in which Ag/AgCl was used as the reference electrode, the platinum plate was the counter electrode, and the glassy carbon (GC, diameter 5 mm) with the applied catalyst as the working electrode. The performed potential measurements were converted in relation to the RHE hydrogen electrode (Reversible Hydrogen Electrode). An ink for electrochemical measurements was prepared by dispersing 2.5 mg of carbon catalyst together with distilled water, ethanol and aqueous Nafion (0.5 wt.% aqueous solution) for 60 min. An appropriate amount of the catalyst was applied to the glassy carbon, and then the solvents were allowed to dry for a few minutes. The results of the catalytic activity were carried out in saturated O_2_ and N_2_ electrolyte of 0.1 M KOH. The catalytic activity of all measured carbon materials was compared to a platinum-based commercial carbon material (20 wt.% Of Pt, Sigma Aldrich) as reference material. Cyclic voltammetry (CV) results were recorded at a 10 mV s^−1^ scan rate and in the potential range from 0 to 0.8 V, while linear sweep voltammetry (LSV) data was recorded at a 5 mV s^−1^ scan rate and a rotating disk electrode (RDE) speed in the range from 800 to 2800 rpm. The number of electrons (n) taking part in the oxygen reduction reaction was calculated on the basis of the LSV plot using the Koutecky–Levich (K–L) formulas presented below:

1 $$ {\text{J}}^{ - 1} = {\text{J}}_{{\text{L}}}^{ - 1} + {\text{J}}_{{\text{K}}}^{ - 1} = \left( {{\text{B}}\omega^{1/2} } \right)^{ - 1} + {\text{J}}_{{\text{K}}}^{ - 1} $$

2 $$ {\text{B}} = 0.62{\text{nFC}}_{0} \left( {{\text{D}}_{0} } \right)^{ - 2/3} \nu^{ - 1/6} $$

The individual parameters of the formula can be defined as J measured current density, J_L_ is assigned to the limiting current density, and J_K_ is defined as the kinetic current density; ω is the angular velocity of the electrode. The value of n, as mentioned above, is the number of electrons involved in the charge transfer in the oxygen reduction reaction; F is defined as a Faraday constant of 96,485 C mol^−1^; C_0_ is the total oxygen concentration in 0.1 M KOH being 1.2 * 10^−6^ mol L^−1^; D_0_ is the oxygen diffusion coefficient in 0.1 M KOH (1.9 * 10^−5^ cm^2^ s^−1^); ν is defined as the kinetic viscosity of the electrolyte used (0.01 cm^2^ s^−1^ for 0.1 M KOH). By determining the slope of the K–L plot, it is possible to estimate the number of electrons transferred in the oxygen reduction reaction.

### Zn–air battery measurements

The Zn–air battery was prepared to characterize the applicability of the obtained materials. The obtained carbon catalyst played the role of a cathode, and the anode was a zinc plate. To assemble the Zn–air battery, you need a microporous membrane (polypropylene membrane, Celgard 5550) and carbon paper with the catalyst applied, as a separator and current collector, respectively. The ink was prepared by dispersing 10 mg of catalyst with 0.9 ml of a mixture of isopropanol and Nafion solutions (5 wt.%, Sigma Aldrich) for 1 h. An appropriate amount of the catalyst was applied to carbon paper (packing 1 mg/cm^2^) and then dried at 80 °C for 30 min. The battery electrolyte was a mixture of 6 M KOH and 0.2 M ZnCl_2_ solutions. All results of galvanostatic charge/discharge (600 charge/discharge cycles − 5/5 min) were carried out on the Autolab potentiostat (PGSTAT302N, the Netherlands). The reference material was an assembled battery with a platinum-based carbon catalyst (20% Pt, Sigma-Aldrich) as the air electrode.

## Results and discussion

### Elemental composition and pore structure

Table 1 presents information on the elemental composition of all investigated materials. The combustion analysis delivers data on the overall elemental content without the split to the surface and bulk composition. The weight share ascribed to the key component, i.e. C, increased upon the rise of carbonization temperature in the range of 700–900 °C. Moreover, the C content was higher than for the precursor GF-750. The difference to 100%, called a residue, decreases in the same order, which means that it was attributed to a removable element as oxygen. GF-750 was a chemically pure material, which contained only minor amounts of durable impurities. Table 2, containing XPS data, confirmed that assumption. Thus, the C-to-O (residue) ratio reached 16.1 for G-900 in contrast to 7.9 for GF-750 (precursor).

**Table 1:** Basic properties of graphene nanoplatelets and electrode materials derived therefrom.

Carbon sample	Elemental content (wt.%) (combustion method)				S_BET_ (m^2^ g^−1^)	S_loop hysteresis_ (cm^3^ g^−1^)	V_t_ (cm^3^ g^−1^)	V_mi_ (cm^3^ g^−1^)	V_me_ (cm^3^ g^−1^)	V_me_/V_t_ (%)
	C	H	N	Residue						
GF-750	87.32	0.90	0.72	11.06	750	9.26	0.999	0.127	0.873	87
G-700	88.81	0.88	0.46	9.85	879	10.36	1.143	0.099	1.044	91
G-800	89.17	0.87	0.53	9.43	805	10.97	1.090	0.104	0.987	91
G-900	93.16	0.71	0.52	5.61	1034	17.05	1.452	0.141	1.311	90

**Table 2:** XPS elemental composition of graphene and electrode materials derived therefrom.

Element	C								O		N	
Binding energy (eV)	284.6	285.0	286.3	287.7	288.6	289.6	292.1		532.0	533.3	400.5	

Carbon sample	Content (at.%)							% of total	Content (at.%)		% of total	Content (at.%)
GF-750	32.6	33.4	9.7	4.4	3.8	6.8	2.6	93.3	2.0	4.8	6.8	0.0
G-700	44.3	28.2	7.5	3.5	1.4	8.0	3.0	95.9	1.3	2.6	3.9	0.3
G-800	45.8	25.9	7.8	3.7	1.8	8.3	3.1	96.4	1.4	2.2	3.6	0.1
G-900	45.2	26.1	8.4	3.7	2.1	8.1	3.1	96.7	0.7	2.6	3.3	0.0

**Figure 1:**
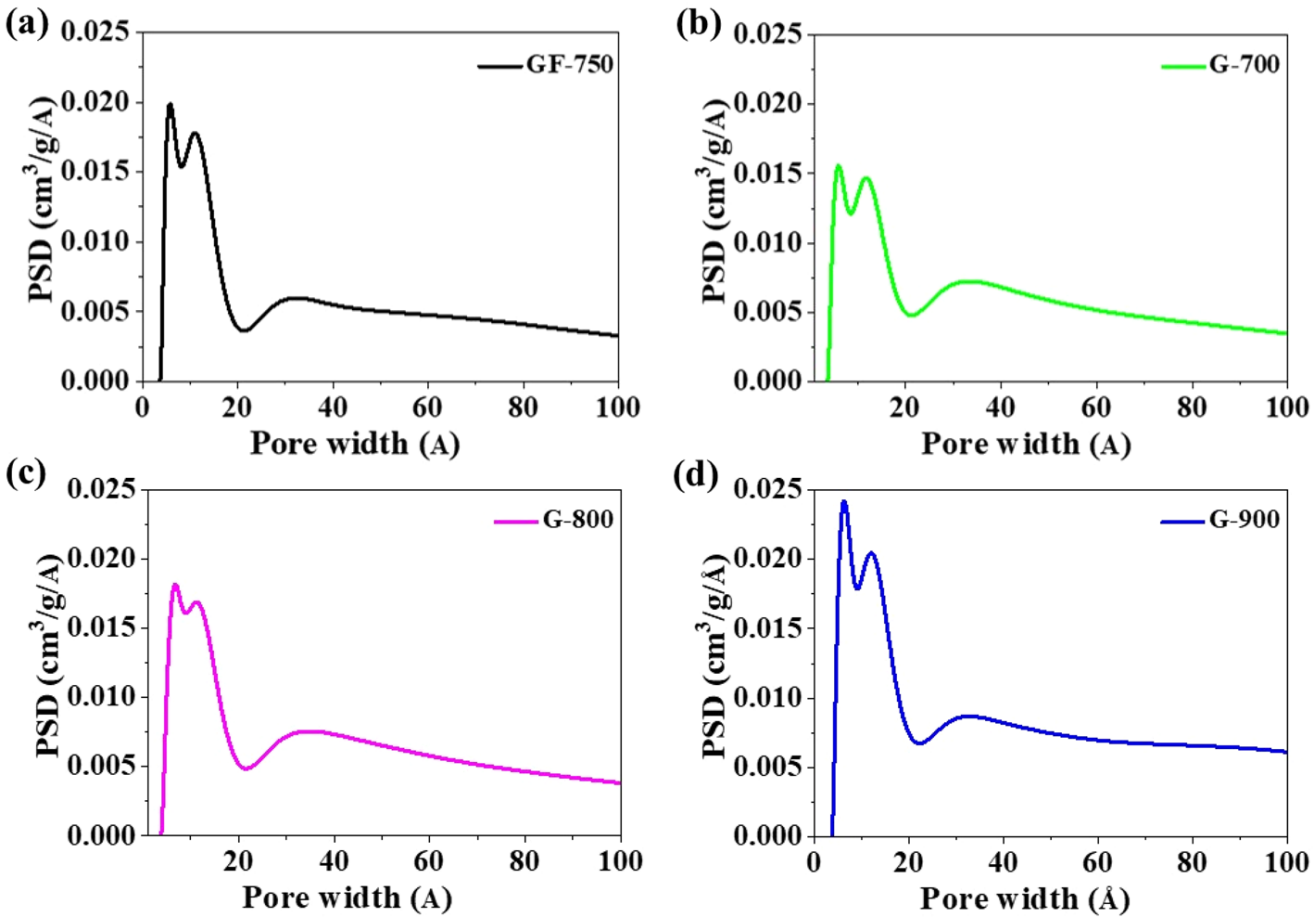
Pore size distribution obtained from adsorption branches of N_2_ used nonlocalized density functional theory (NLDFT) method of samples (**a**) GF-750, (**b**) G-700, (**c**) G-800 and (**d**) G-900.

**Figure 2:**
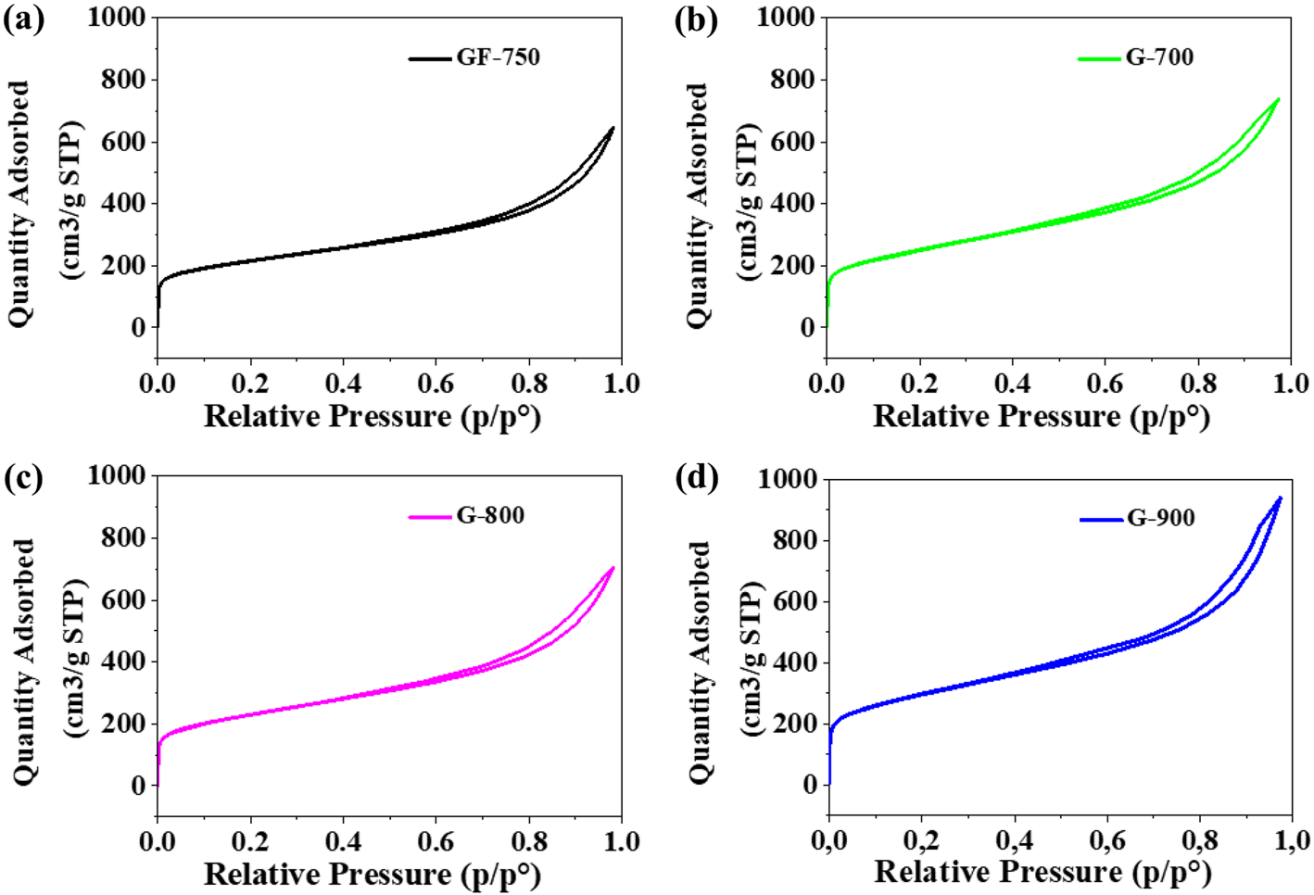
Nitrogen adsorption–desorption isotherms of samples (**a**) GF-750, (**b**) G-700, (**c**) G-800 and (**d**) G-900.

In general, G-materials are more “pure” regarding the content of elemental carbon than the precursor GR-750. The effect is understandable since all G-materials were subjected to intensive thermal elaboration, which obviously led to the elimination of heteroatoms (mainly oxygen) due to the thermal destruction of oxygen species bonded to graphene planes. Figure 1 demonstrates the pore size distribution obtained from the nonlocalized density functional theory (NLDFT) method in the SAIEUS program (Carbon-N2, NLDFT, Standard Slit model). The results confirm the presence of mesopores in the structure of the obtained materials. It is a micro-mesoporous material with a predominant amount of micropores. The pore size distribution shapes indicate that the obtained materials have a similar porous structure. On the other hand, in Fig. 2, the shapes of the hysteresis loops indicate the low presence of mesopores^25^. The hysteresis loops area confirms that the number of mesopores slightly increases with increasing temperature carbonization of the sample. According to the IUPAC classification, all nitrogen adsorption–desorption isotherms for the obtained materials are type II.

Table 1 illustrates the surface area improvement in the case of all G-samples in comparison to the precursor, i.e. nanoplatelets. The increase is particularly spectacular (ca. 45%) for G-900, the material heat-treated at the highest temperature. In parallel, one observed a change of the pore structure, i.e. the total pore volume raised from 0.999 (GF-750) to 1.452 cm^3^/g (G-900). The contribution of the mesopore volume V_me_ to the total pore volume V_t_ also increased from 87 to 91% for all G-samples. All structural improvements, i.e. surface area, total pore volume and the volume share ascribed to mesopores, are particularly expected assuming that the G-materials are expected to be effective electrodes for ORR. A well-developed surface area positively influences the numbers of catalytic centres, while a well-developed and adequately tailored pore structure positively influences molecular transport to and off the electrode surface (reduction of electrode polarization). In summary, the applied manufacturing protocol led to positive structural changes compared to pristine GF-750 graphene nanoplatelets.

The XPS data (Table 2) prove that the surface of the investigated G-materials is composed of carbon atoms (atomic content from 93.3 to 96.7%) and heteroatoms as O atoms rarely occur (atomic content from 3.3 to 6.8%). The occurrence probability of N atoms is marginal since the atomic N content does not exceed 0.3 at.%. Literature commonly attributes high activity N-doped carbon electrode materials in ORR to the presence of nitrogen atoms^26–29^. N atoms are seen as catalytic centers replacing Pt-based catalytic centers as in the commercial reference material denoted as Pt/C in this study. Therefore, the observed high electrochemical ORR activity of the G-materials cannot be attributed to the presence of N atoms and must result from other factors like the improved pore structure and surface area, as proved in the subsequent chapters.

### Structure

**Figure 3:**
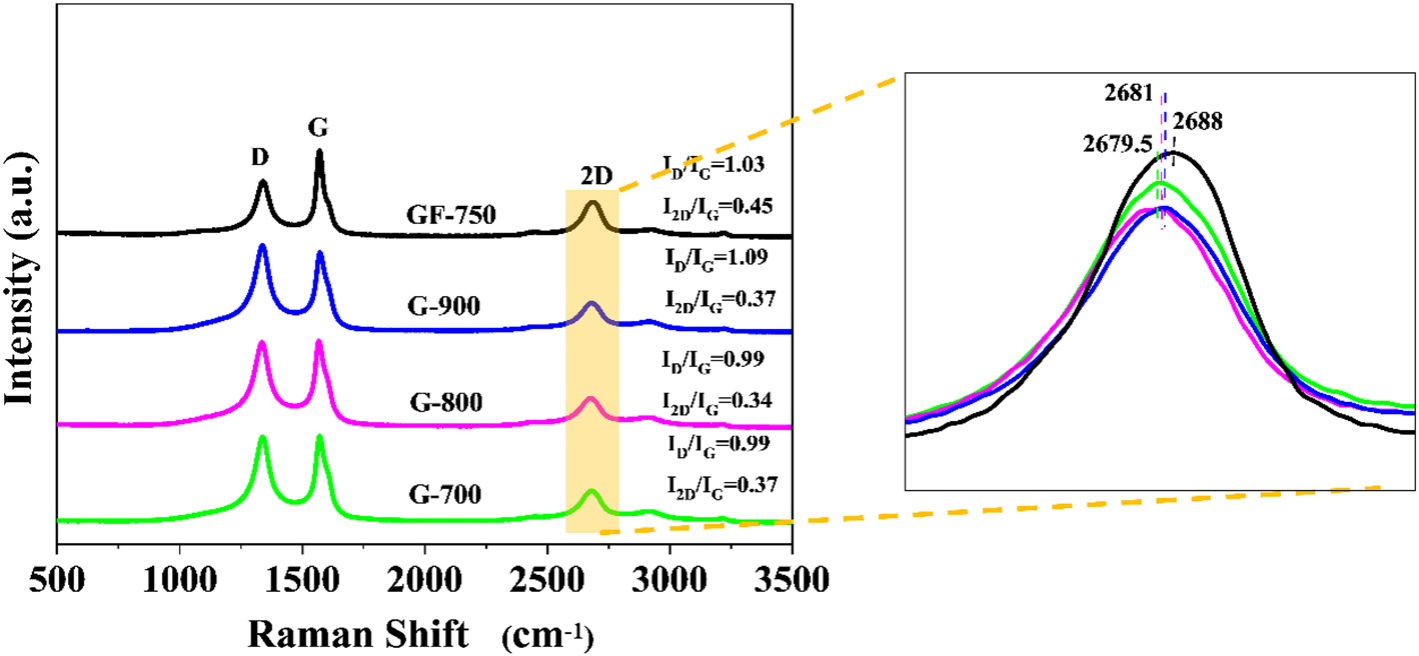
Raman spectra of graphene-based electrode materials.

As mentioned, the manufacturing procedure assumed that graphene planes stacked into bigger blocks were first fragmented to less complex structures (due to the high-pressure liquid phase method), then reorganized, and finally fixed into a 3D porous structure. Thus, single graphene layers (SLG) or few-layered graphene (FLG) were expected to be a building stone of the final structure. Raman spectroscopy studies were performed to investigate this issue. Table 3 and Fig. 3 contain information on the D, G, 2D band placement and intensity. A shift of the G band towards higher frequencies would have meant a diminishing of the graphene plane agglomeration degree in the thereof materials. However, the differences were not systematic and spectacular, which means that similarly, agglomerated graphene blocks were present in each investigated material, including GF-750. The I_D_/I_G_ ratio did not change considerably, too. This means that the size of crystalline domains and the number of defects were comparable for all electrode material samples and GF-750. The intensity ratio I_2D_/I_G_ and I_D_/I_G_ of investigated samples indicate that the obtained samples are consisting the multilayer nature of the graphene. The Raman data allowed us to conclude that the liquid phase splitting of GF-750 dispersed graphene packages existing GF-750 in the liquid phase rather than intensively splitting the packages to SLG. The carbonization temperature of the samples affects both the position of the G and 2D bands. G-900 sample had more vacancies and disorders since it has the highest I_D_/I_G_ ratio and the highest carbon at.%^30^. However, the G band position of G-900 was in a higher wavenumber compare to G-700 or G-800 sample. The observed 2D band intensity decreases with increasing numbers of graphene layers and carbonization range^31^.

**Table 3:** D, G and 2D band data of Raman spectra.

Sample	I_D_	cm^−1^	I_G_	cm^−1^	I_2D_	cm^−1^	I_D_/I_G_	I_2D_/I_G_
GF-750	1.00	1344.5	0.97	1578.0	0.44	2679.0	1.03	0.45
G-700	0.99	1338.5	1.00	1571.0	0.37	2679.5	0.99	0.37
G-800	0.99	1336.5	1.00	1568.5	0.34	2680.0	0.99	0.34
G-900	1.00	1337.5	0.92	1573.0	0.34	2681.5	1.09	0.37

**Figure 4:**
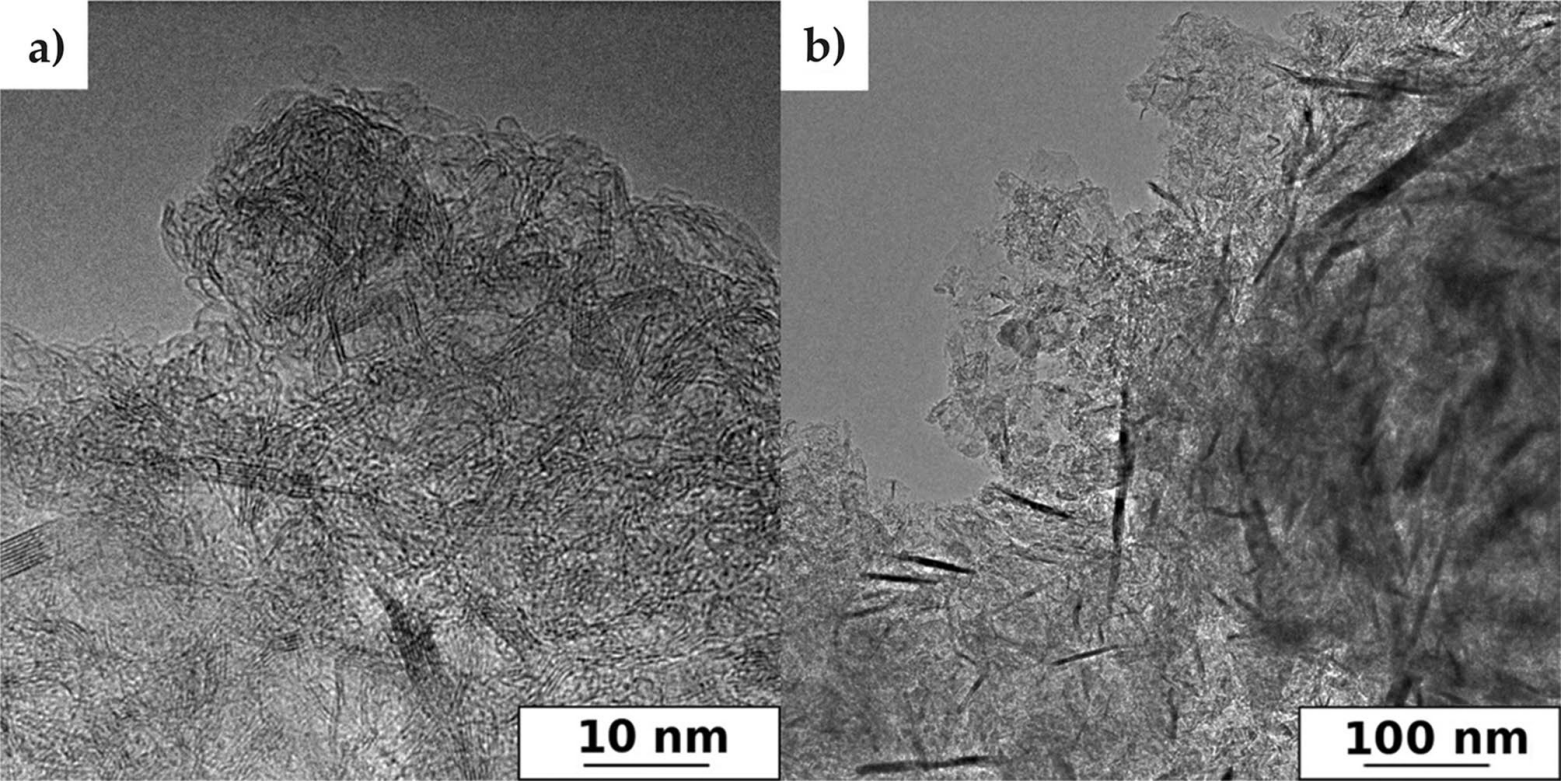
HRTEM images of graphene-based electrode materials. FLG (below 10 layers) packages detected in G-700 (left—high resolution) and G-800 (right—low resolution).

**Figure 5:**
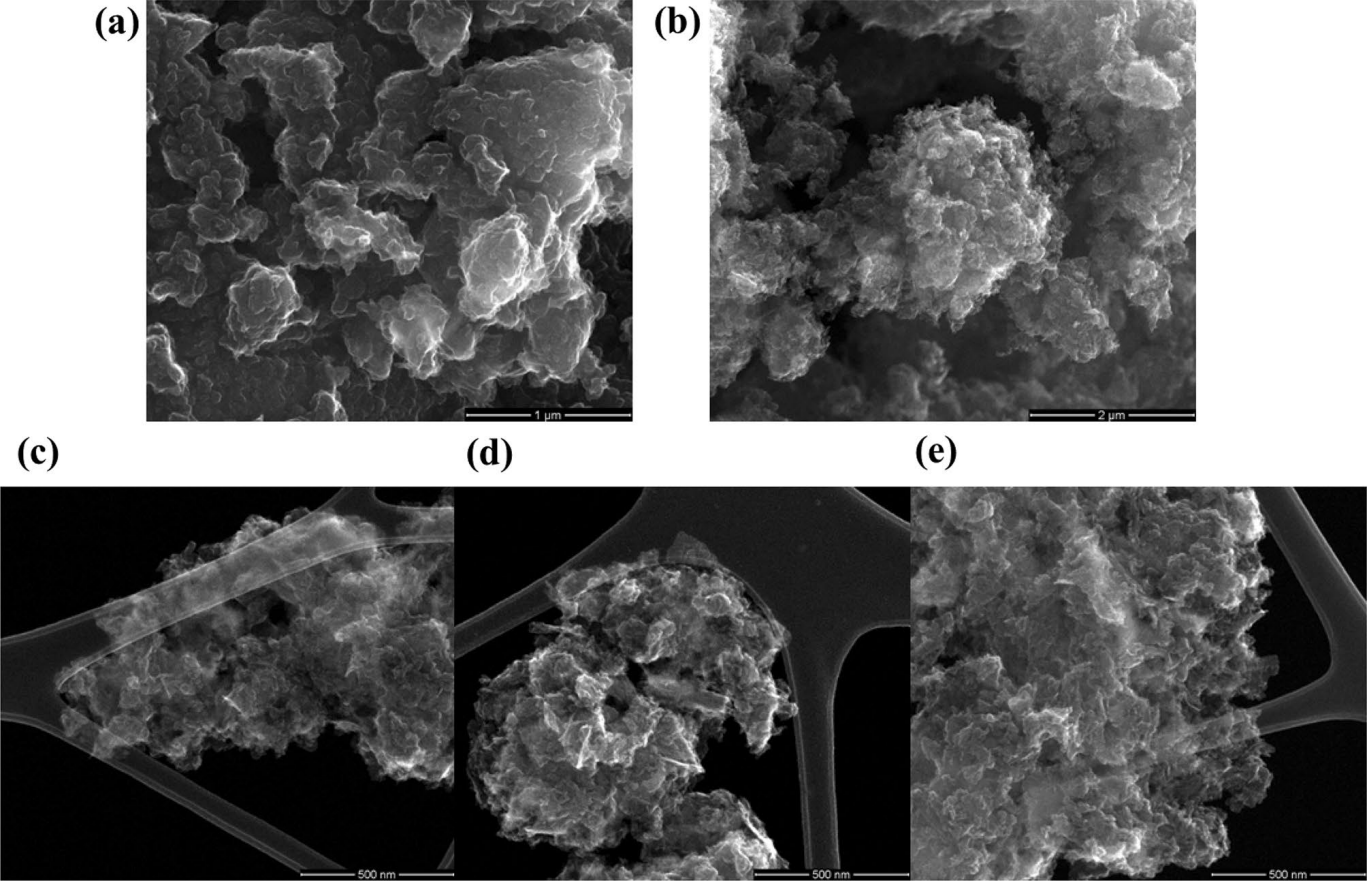
SEM images of samples (**a**) GF-750, (**b**) G-800, and in the STEM mode (**c**) G-700, (**d**) G-800 and (**e**) G-900.

The exfoliation to FLG, although not fully confirmed by the Raman studies, was proved by HRTEM structural investigations. Figure 4 documents that FLG structures are definitely present in the electrode materials, in contrast to GF-750, in which they occur rarely. Figure 4 (right) demonstrates the presence of dark “dashes”, which are edges of FLG oriented perpendicular to the observation point. A high-resolution image, i.e. Figure 4 (left), confirms this since the “dashes” are built of few parallel stack individual graphene planes. Additionally, it can be concluded that the analyzed materials are pure without a residue of the used template. Thus, the HRTEM and Raman investigations are consistent as for the structure of the investigated samples. In turn, Fig. 5 presents SEM images of obtained materials. All samples had similar irregular and porous surface characteristics, are good quality and again confirm the purity without a residue of the used CaCO_3_. These images showed a dense 3D pore structure of materials. Generally, the SEM images do not fully allow to determine the degree of graphene sheets deglomeration in these 3D structures.

### Surface chemistry

**Figure 6:**
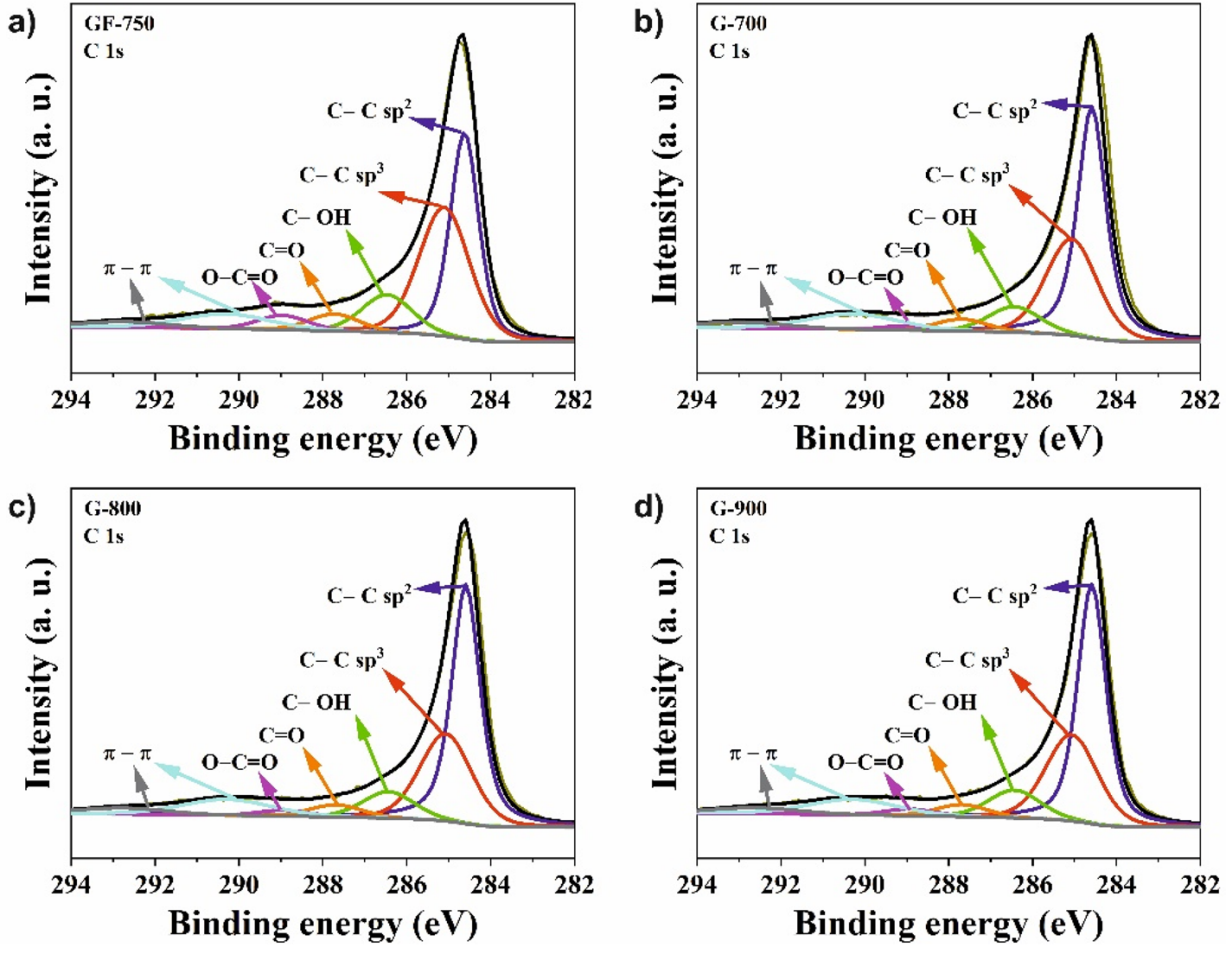
High-resolution X-ray photoelectron spectra for C1s of all investigated samples: (**a**) C1s of GF-750, (**b**) C1s of G-700, (**c**) C1s of G-800, and (**d**) C1s of G-900.

**Figure 7:**
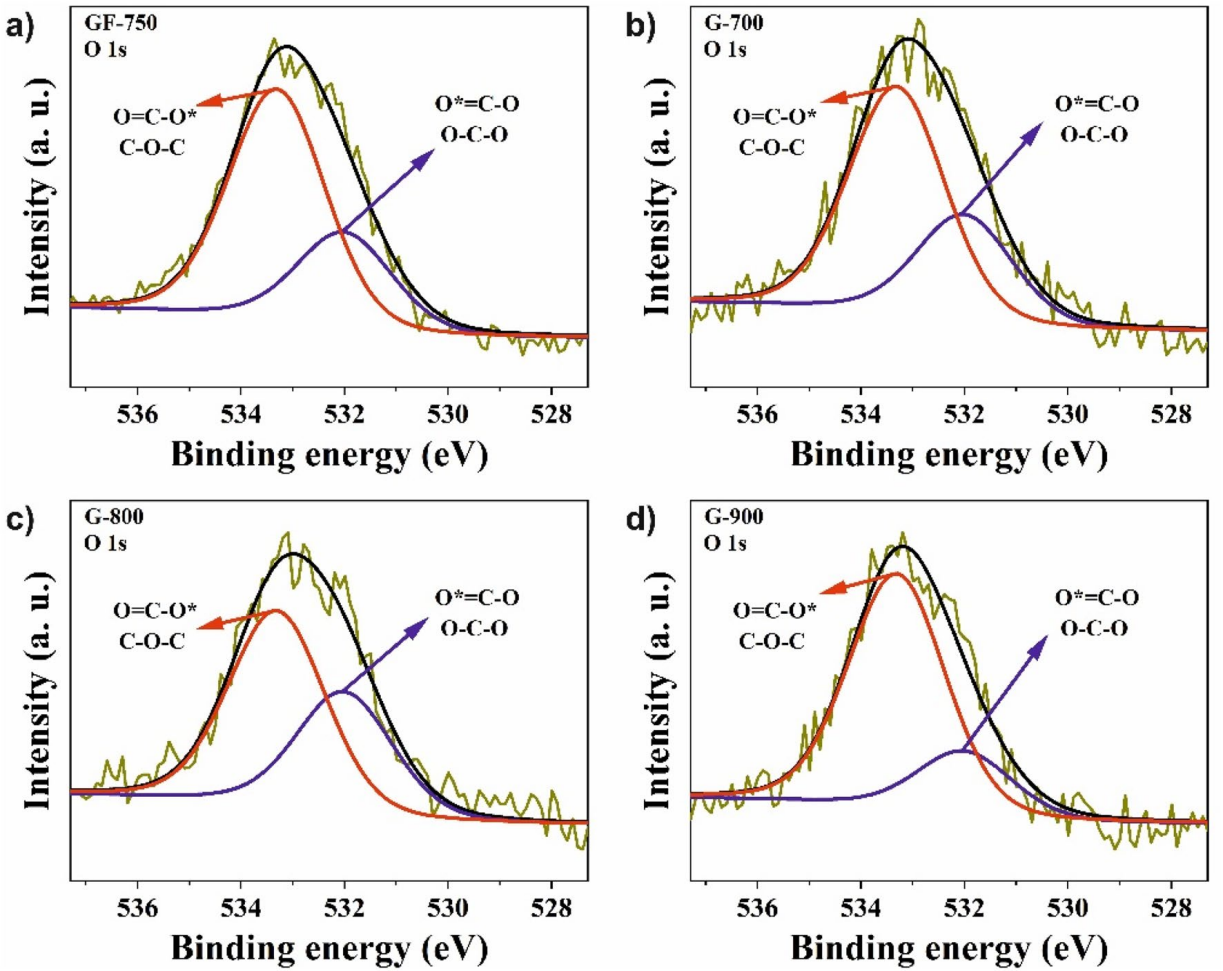
High-resolution X-ray photoelectron spectra for O1s of all investigated samples: (**a**) O1s of GF-750, (**b**) O1s of G-700, (**c**) O1s of G-800, and (**d**) O1s of G-900.

High-resolution XPS studies were performed to determine the chemical structure of the investigated G-materials and pristine GF-750 graphene. Surface elemental composition data are presented in Table 2. The XPS spectra of all investigated samples are demonstrated in Figs. 6 and 7. The analysis of the C1s energy is essential in determining the chemical bonding of carbon atoms. The elemental content of the obtained carbons was high, ranging from 95.9 to 96.7 at.%. In comparison to raw material, GF-750 is observed increased carbon content of about 2.6–3.4 at.%. Carbon atoms were mostly bonded as sp^2^ hybridized atoms (band C 1 s at a binding energy of 284.6 eV), which is characteristic of graphene materials. The C1s spectra of the obtained materials are composed of seven peaks corresponding to C=C bond (sp^2^) peak at 284.6 eV^32^, C–C bonds (sp^3^) peak at 285.0 eV^33^, C–O–C or C–OH bond peak at 286.3 eV^34^, C=O or O–C–O bond peak at 287.7 eV^32^, and peaks with binding energy at 289.6 eV and 292.1 eV are associated with shake-up excitation^32^. The total amount of oxygen in the obtained samples is in the range of 3.3–3.9 at.%, respectively. In turn, the raw material GF-750 has 6.8 at.% of oxygen.

### Electrochemical performance

**Figure 8:**
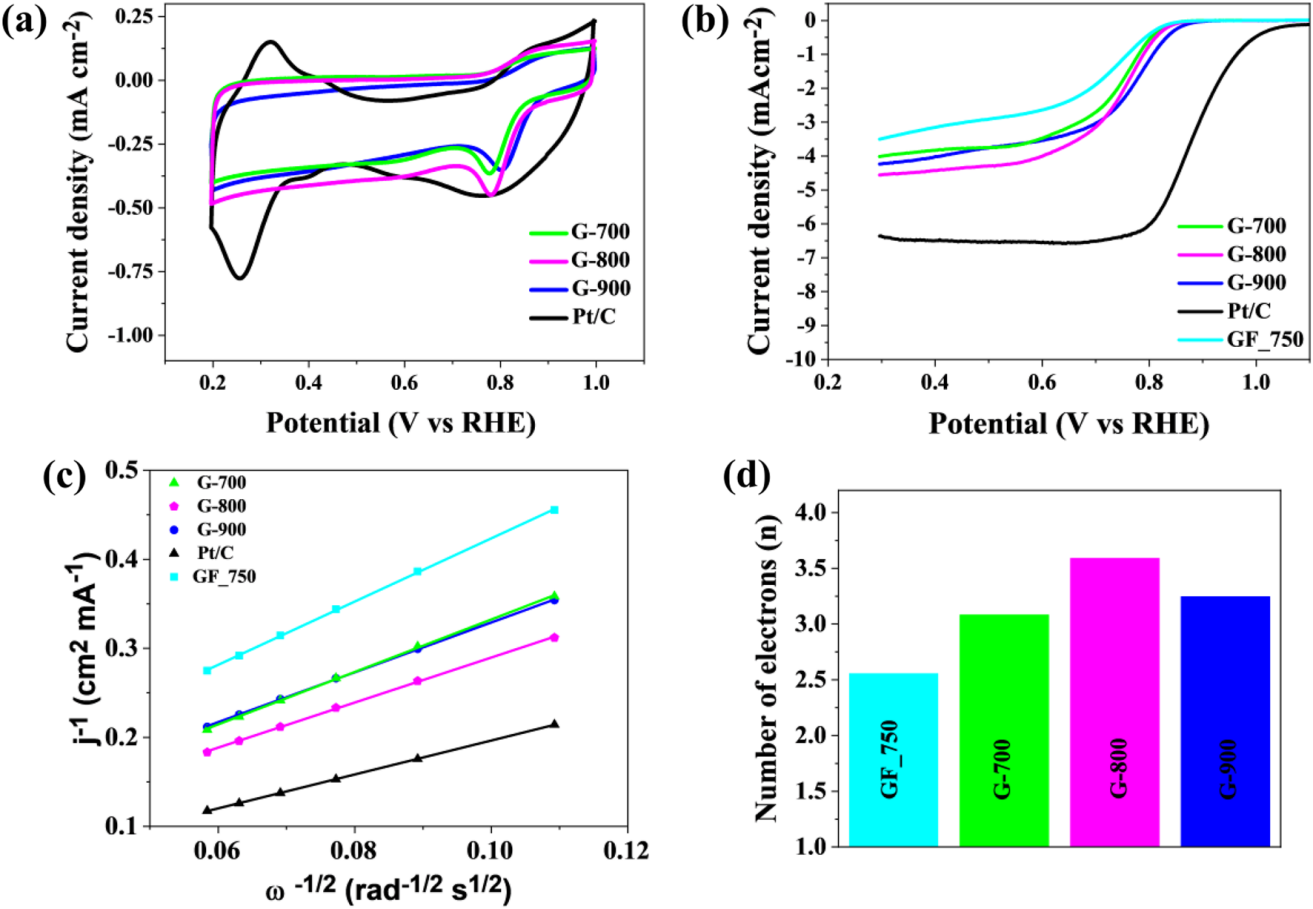
(**a**) CV curves of the obtained electrocatalysts and Pt/C catalysts in an O_2_-saturated 0.1 M KOH solution; (**b**) LSV curves of G-700, G-800, G-900 and Pt/C catalysts measured at a scan rate of 5 mV s^−1^ and a rotation rate of 1600 rpm in O_2_-saturated 0.1 M KOH solution; (**c**) Koutecky–Levich plots in the potential 0.5 V; (**d**) the number of transfer electrons in the oxygen reduction reaction.

The catalytic activity of carbon materials was investigated using the techniques of cyclic voltammetry (CV) and linear sweep voltammetry (LSV). All measurements were made in 0.1 M KOH saturated with oxygen and nitrogen. The voltammogram in Fig. 8a shows the CV curves from which a clear cathode peak (Ep) is visible in all the obtained graphene materials. The cathode peak is clear for all samples. Table 4 shows the oxygen reduction reaction parameters necessary to characterize the catalytic activity of the electrocatalyst. The value of the cathode peak for commercial platinum-based carbon (Pt/C, 20% wt.) is 0.76 V versus RHE, while for the samples, clear peaks are observed for G-700, G-800, G-900 versus RHE, amounting to 0.79 V, 0.78 V, 0.78 V and 0.80 V, respectively. Further linear sweep voltammetry (LSV) measurements were performed with a rotating disc electrode (RDE) in the rpm range of 800–2800 in an O_2_ saturated electrolyte of 0.1 M KOH. Table 4 shows the starting potential values ​​for each sample at 1600 rpm resulting from the linear RDE sweep (Fig. 8b).

**Table 4:** ORR performance parameters of the obtained reactor carbon and commercial Pt/C catalysts tested in alkaline media.

Catalysts	E_p_ (V vs. RHE)	E_onset_ (V vs. RHE)	E_1/2_ (V vs. RHE)	Diffusion-limiting current (mA cm^−2^)	n (0.5 V)
Pt/C (20% wt.)	0.76	1.03	0.88	6.37	4.00
GF-750	0.77	0.821	0.73	2.78	2.56
G-700	0.78	0.818	0.75	3.73	3.08
G-800	0.78	0.823	0.75	4.25	3.59
G-900	0.80	0.846	0.78	3.46	3.25

Szczesniak et al.^35^ proved that in carbon materials, some intrinsic structures as armchair edges and zigzag edges could strongly interact with neighbouring carbon atoms and change their electronic structure similar to the inserted heteroatoms doping in several different configurations could also significantly alter the electron distribution of dopants and adjacent C atoms via the delocalization of π electrons, thereby affecting the physical and chemical properties. Similarly, constructing a vacancy/defect by removing a C atom may not only destroy the unity of π conjugation but also exert a significant influence on the electronic structure of adjacent C atoms.

Nørskov et al.^36^ defined a possible ORR mechanism involving two key stages: an associative mechanism involving a HOO* species and a direct O_2_ dissociation mechanism in an acid or alkaline electrolyte. In an alkaline environment, the following steps may occur:

3 $$ O_{2 \left( g \right)} + * \to O_{2}^{*} $$

4 $$ O_{2}^{*} + H_{2} O \left( I \right) + e^{ - } \to HOO^{*} + OH^{ - } $$

5 $$ HOO^{*} + e^{ - } \to O^{*} + OH^{ - } $$

6 $$ O^{*} + H_{2} O \left( I \right) + e^{ - } \to HO^{*} + OH^{ - } $$

7 $$ HO^{*} + e^{ - } \to OH^{ - } + * $$

The mechanism via direct O_2_ dissociation of oxygen atoms starts with the following elementary step:

8 $$ O_{2 \left( g \right)} + * + * \to O^{*} + O^{*} $$

followed by steps (6) and (7) in alkaline solution.

Where * stands for an active site on the surface of electrocatalyst, ($I$) and (g) refer to liquid and gas phases, respectively, and O*, OH* and HOO* are the adsorbed intermediates.

Splitting of graphene flakes contributes to the enlargement of specific surface areas and opens active sites such as carbon atom vacancies, which are less accessible in the case of stacked graphene sheets. We assume it is the main reasoning for the observed increase of ORR activity. The number of transferred electrons in ORR, i.e. n, is the highest for G-800 and G-900, also having the most advanced structural parameters as surface area and the total pore volume.

The ORR highest-performing material of the investigated graphene-based electrodes is G-900, which catalyzes the ORR at potentials within 160 mV of the paternal Pt/C electrode for a current of 1 × 10^−2^ mA cm^−2^. However, the differences between G-900 and G-800/G-700 are visible but not spectacular. This is in contrast to the pristine GF-750 sample, which performs ORR much worse. Using the Koutecky–Levich diagram (Fig. 8c), it is possible to determine the number of transferred electrons in the oxygen reduction reaction at the value of 0.5 V. The results for each catalyst obtained in the speed range (800–2800 rpm) showed a linear character, which indicates the correct spread of electron transfer in the ORR reaction. The number of transferred electrons (Table 4 and Fig. 8d) for most of the obtained carbon materials is above 3. The highest value of the number of electrons transferred for the G-800 sample is 3.6, which indicates a similar 4-electron oxygen reduction reaction as in the case of a commercial catalyst. The obtained materials, despite the low nitrogen content in the structure, show increased catalytic activity. As demonstrated in our previous work^37^ electrode material properties are predominantly affected by their pore structure, carbonization temperature, modification of structure and the presence of heteroatoms. Not all of these critical parameters have a positive effect on the electrochemical performance of the carbons in ORR. The high surface area and porosity of the obtained graphene materials allow for unforced access of the KOH electrolyte into the porous structure, increasing the catalytic activity. Therefore, these materials can be successfully used as metal-free catalysts in devices based on the oxygen reduction reaction, such as metal-air batteries or fuel cells. The porosity of the graphene structure has a significant impact on the catalytic activity, and the appropriate pore size means that materials with a high surface area can also be used in energy storage devices. For the sample with the highest surface area, the applicability of the obtained materials was tested as a Zn–air battery.

The improvement of the ORR performance of G-materials against pristine GF-750 is spectacular, i.e. the key parameter “n” increased from 2.56 to 3.59 in the case of G-800. It has to be noted that the efficient ORR performance was achieved due to 3D structuring (improvement of surface area and pore structure), while the chemical purity was much better for G-materials. Therefore, the high ORR activity of G-materials should not be ascribed to heteroatom catalytic centers since such heteroatoms were successively removed upon increasing the carbonization temperature. The XPS data showed that the presence of transition metals (usually regarded as catalytic centers) in G-materials was marginal. Also, the content of nitrogen atoms potentially being active in ORR was neglectable. The results point out the importance of the structural factors of electrodes in the case of graphene-based materials and ORR.

**Figure 9:**
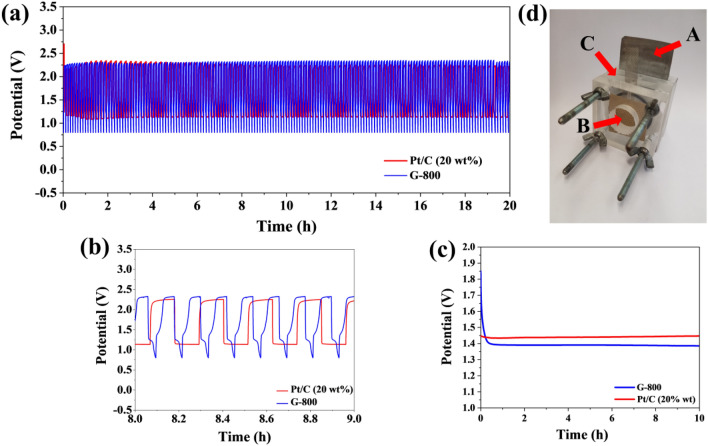
(**a**) Galvanostatic charge/discharge cycling curves at 1 mA cm^−2^ of Zn–air batteries incorporating the G-800 catalyst and the commercial Pt/C catalyst, respectively; (**b**) galvanostatic charge/discharge cycling in the selected range; (**c**) galvanostatic discharge curves at 1 mA cm^−2^ for G-800 catalysts and the commercial Pt/C catalyst; (**d**) photograph of the handmade rechargeable Zn–air battery with an open-circuit voltage of 1.38 V, where (**A**) Zn electrode, (**B**) air electrode (GDL made of the catalyst under investigation), (**C**) electrolyte 6 M KOH with 0.2 M ZnCl_2_.

Based on the results of the oxygen reduction reaction, the material with the highest transferred electron number was selected and used as an oxygen catalyst in the construction of the metal–air battery. The purpose of this study was to check the applicability of the obtained graphene materials. Zn–air batteries were self-designed and handmade (Fig. 9d). For this purpose, a zinc plate was used as an anode electrode, separator and the above-mentioned carbon paper with a charged catalyst with a packing of 1 mg/cm^2^. The charge/discharge tests for the best catalyst were compared to a metal-air battery made of a platinum-based carbon catalyst (Pt/C 20 wt.%). Three hundred charging/discharging cycles were performed (one cycle consisted of 300 charges and 300 discharges). The stability of the open circuit potential (OCP) confirms the effective operation of Zn–air batteries 1.38 V. Compared to the Pt/C catalyst, whose potential was 1.45 V, the potential for the catalyst obtained with our method was close to this value. It can be assumed that the excellent charge/discharge stability, shown in Fig. 9, was the presence of a high surface area, a mesoporous structure responsible for the catalytic activity. The charge/discharge tests were conducted at a current density of 1 mA/cm^2^. They showed stability in the range of 2.27 V for a charged battery and 0.9 V for the discharged process. The potential range for Zn–air batteries with a G-800 catalyst is stable over a long period of time. After 6 h of charge/discharge cycles, the potential of the battery with the commercial catalyst falls, while the battery with the metal-free catalyst keeps its charging stability throughout the charging/discharging process. The obtained mesoporous graphene material can be successfully used as electrode material in devices using the oxygen reduction reaction, and due to the appropriate specific surface area and pore size, the use of this material can be extended for application in supercapacitors.

## Conclusions

In summary, we have demonstrated a low-cost method to obtaining high surface area mesoporous graphene materials. We have shown that the porous structure of the material is of key importance in electrochemical applications. The obtained mesoporous graphene material can be successfully used as electrode material in devices using the Oxygen Reduction Reaction, and due to the appropriate specific surface area and pore size, the use of this material can be extended for application in supercapacitors. These aspects of the performed study will be continued in further research.
